# Adult Type II diastematomyelia with tethered cord and associated spinal anomalies: A case report

**DOI:** 10.1016/j.radcr.2025.08.036

**Published:** 2025-09-11

**Authors:** Sachchu Thapa, Bijay Kunwar, Anup Ghimire, Atul Mainali, Pujan Pandey, Jagdish Kunwar, Roshna Adhikari

**Affiliations:** a Department of Radiodiagnosis, National Academy of Medical Sciences, Bir Hospital, Kathmandu, Nepal; b Tribhuvan University Institute of Medicine, Maharajgunj Medical Campus, Kathmandu-3, P.O. Box: 1524, Kathmandu District, Kathmandu, Bagmati, Nepal; c Department of Pediatrics, National Academy of Medical Sciences, Kanti Children’s Hospital, Kathmandu, Nepal

**Keywords:** Diastematomyelia, SCM, Tethered cord, Spinal dysraphism, Hemimyelocele, Dorsal dermal sinus

## Abstract

Spinal dysraphism consists a group of congenital anomalies due to defective neural tube closure, among which diastematomyelia or split cord malformation is rare. Split cord malformation is classified into Type I and Type II, with Type II being less common and often asymptomatic. These anomalies may coexist with various spinal anomalies, as tethered cord, neural lipoma, hemimyelocele, and dorsal dermal sinus, forming a complex spectrum. Adult presentations are particularly uncommon and usually incidental. We herein report a 23-year-old male with chronic low back pain and a congenital midline lumbar swelling. Neurological examination and routine investigations were unremarkable. Magnetic resonance imaging revealed Type II diastematomyelia with two hemicords within a single dural sac from L4 to L5, low-lying conus medullaris, hemimyelocele with neural lipoma at L5-S1, tethered cord with filum terminale lipoma extending to S3-S4, and a dorsal dermal sinus tract. Despite the radiological complexity, the patient remained neurologically intact. Neurosurgical intervention was advised; however, the patient chose conservative management with close follow-up. This case highlights a rare adult presentation of complex spinal dysraphism with minimal symptoms. While diastematomyelia Type II is typically diagnosed in childhood, adult cases are infrequent and often delayed. Magnetic resonance imaging is crucial for diagnosis, particularly in asymptomatic individuals with cutaneous markers. Though surgery is generally recommended to prevent neurological decline, conservative management may be appropriate in selected stable cases. This case emphasizes the need to consider spinal anomalies in adults with chronic back pain and the importance of multidisciplinary evaluation.

## Background

Spinal dysraphism encompasses a spectrum of congenital anomalies resulting from defective neural tube closure during early embryogenesis. Diastematomyelia, or split cord malformation (SCM), is a rare subtype defined by sagittal division of the spinal cord into two hemi-cords separated by a fibrous, cartilaginous, or osseous septum [1,2]. According to Pang’s classification, SCM is divided into Type I—characterized by hemi-cords in separate dural sacs with a rigid osseocartilaginous spur—and Type II, where both cords share a single dural sac divided by a nonrigid fibrous septum [3]. Type II SCM is less common and often underdiagnosed due to subtle clinical and radiologic findings [4].

SCM is frequently associated with other anomalies such as neural lipomas, tethered cord syndrome (TCS), dermal sinuses, and scoliosis [1,4]. Neural lipomas result from premature disjunction of cutaneous ectoderm from the neural tube, leading to incorporation of adipose tissue into the neural axis [5], and may contribute to cord tethering and neurological deterioration [6]. TCS, marked by abnormal fixation of the spinal cord, causes progressive traction-induced ischemia and presents with motor, sensory, and sphincter dysfunctions [7].

The coexistence of Type II SCM with TCS, filum terminale lipoma, hemimyelocele, and dorsal dermal sinus is exceptionally rare, with few reported cases in literature [4,8]. Magnetic resonance imaging (MRI) remains the diagnostic modality of choice, offering superior delineation of spinal anatomy critical for preoperative planning [4,9].

We report an adult case with this constellation of findings, emphasizing its diagnostic challenges and the importance of a multidisciplinary approach for comprehensive evaluation and management.

## Case presentation

### Clinical presentation

A 23-year-old male presented with swelling over the lower back region since birth and chronic low back pain with increasing intensity for a month. Pain is a dull aching, increasing in intensity (7/10 in visual analogue scale) in the lower back for the past 1 month. The pain is localized to the lumbar region, nonradiating, and not associated with any aggravating or relieving factors. He complains of the presence of a swelling over the lower back, which is fleshy (fatty subcutaneous masses) since birth. The swelling has remained relatively unchanged in size over the years. There is no history of discharge, ulceration, or infection at the site. There was no history of tingling or burning sensations in limbs, weakness, or bowel and bladder involvement. There are no constitutional symptoms such as weight loss or appetite change. His medical, surgical, and birth developmental history was unremarkable.

**Fig. 1A-G:**
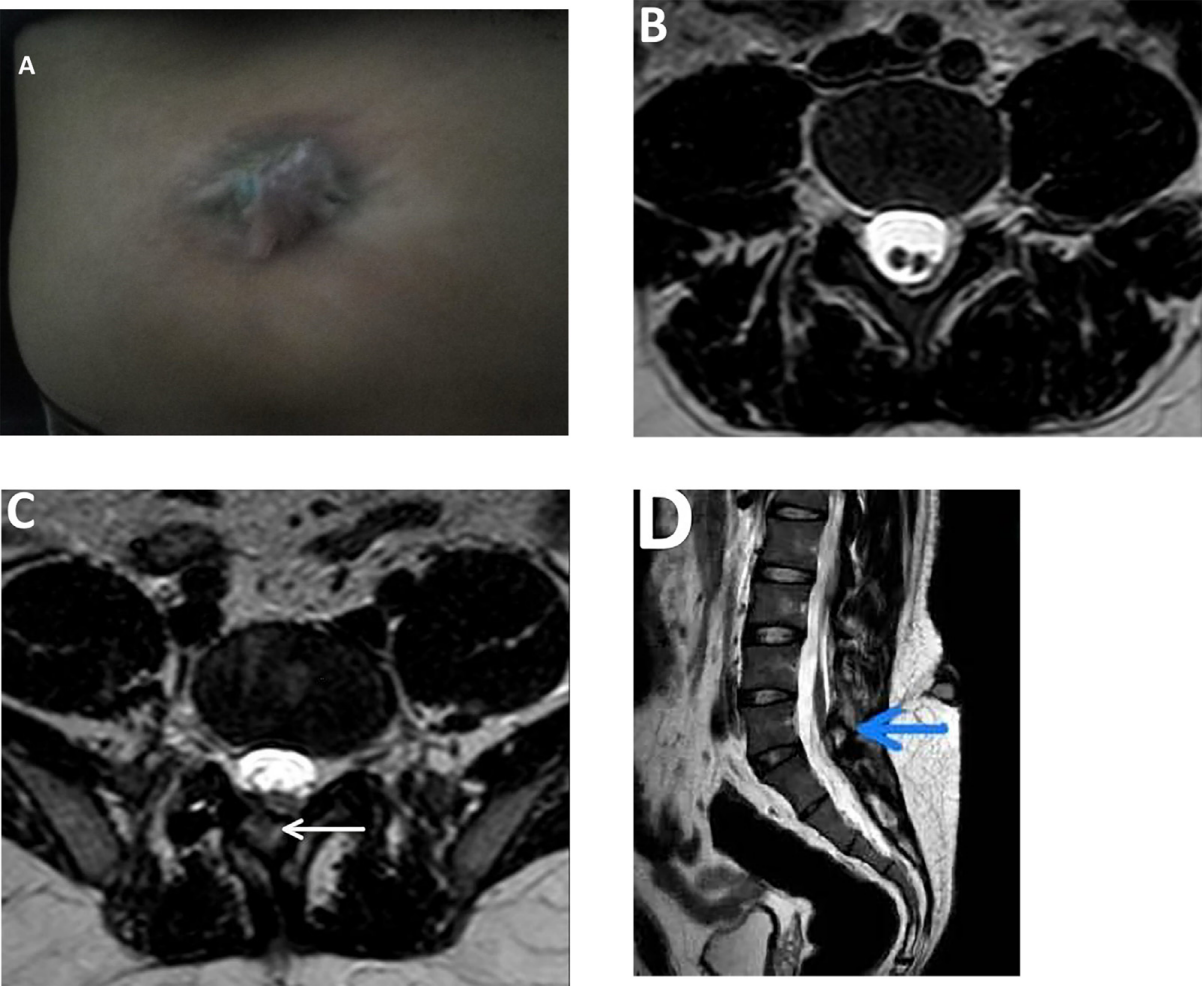

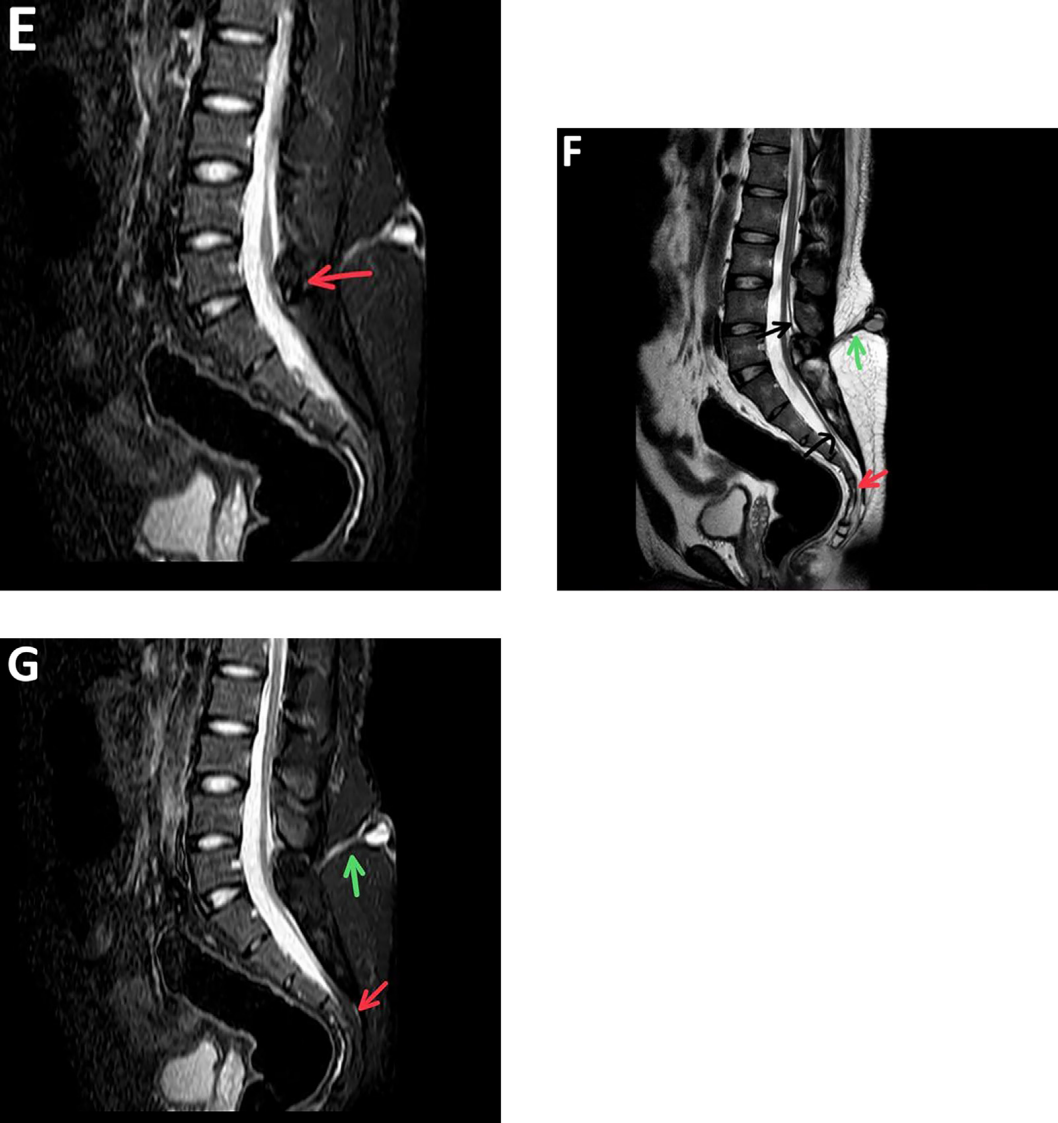
(A) Clinical photograph of the lower back (lumbar region) showing a midline reddish-fleshy lesion without audible bruit, suggestive of an angiomatous skin lesion associated with a dorsal dermal sinus. (B) T2 weighted axial view showing hemi-cords within a single dural sac without spur and septum; from lower border of L4 to lower border of L5 vertebral level−diastematomyelia Type II. (C) T2 weighted axial at L5-S1 level and (D) T2 weighted sagittal and (E) STIR sagittal view image showing neural-lipoma, hemi placode (as shown by arrows) is extended within the posterior bony defect (not extending beyond skin surface) at L5-S1 level. (F) T2 weighted axial and (G) STIR sagittal view shows conus medullaris extending below the level of L2 vertebra, projecting all the way to S3-S4 level (one hemi cord) and L5-S1 level (other hemi cord with spina bifida) as shown by the black arrow. At S4 level, the hemi cord is attached to an attenuating mass showing T2 high signal intensity, which is suppressed on STIR image shown by red arrows, suggestive of tethered cord with film terminale lipoma. A linear T2/STIR high signal intensity tract is noted extending from the skin surface to the bony defect at L5-S1 level, shown by green arrows, suggestive of a dorsal dermal sinus.

On examination, the patient is conscious, alert, and oriented. His vital signs were stable, and general examination was unremarkable. Local examination: About 2 × 2 cm reddish bluish fleshy midline swelling noted over the lumbar region, shown in Fig. 1A. It is nontender, soft in consistency. There was no visible sinus or discharge, signs of infection, bruit on auscultation, or local rise of temperature. Neuro examination was unremarkable. Motor examination revealed symmetrical muscle bulk, tone was equal and symmetric in all flexor and extensor groups of muscles. Muscle power was 5/5 in both the upper and lower extremities. Bilateral deep tendon reflexes were normal and equal. Clonus was absent. Systemic examination revealed no abnormalities.

### Investigations

The laboratory exams of the patient (CBC, vitamin D3, ESR, CRP, LFT, RFT, HLA-B27) were within normal ranges. A tuberculin skin test was negative. The x-ray of the Lumbosacral spine was normal. With this, all the common differentials of back pain, mechanical, infectious, inflammatory, and malignancy, were ruled out, and an MRI was planned for the whole spine.

MRI revealed the following findings: Axial T2-weighted images demonstrated two hemi-cords within a single dural sac extending from the lower border of L4 to the lower border of L5 with no evidence of osseous or fibrous spur or septum consistent with Type II diastematomyelia (Fig. 1B). A T2 hyperintense, fat-signal lesion is seen extending into a posterior bony defect at the L5-S1 level, representing a neural lipoma (Fig. 1C-1E). The hemi-placode is seen extending into this defect, but there is no cutaneous extension (Fig. 1C-1E). The conus medullaris is low-lying, with one hemi cord extending to the S3-S4 level, and the other terminating at the L5-S1 level, associated with spina bifida (Fig. 1F and 1G). At S4, the hemi cord is seen attached to a T2 hyperintense, STIR-suppressing fat-containing lesion, consistent with a filum terminale lipoma and tethered cord (Fig. 1F and 1G). A linear hyperintense tract on T2 and STIR sequences is noted, extending from the skin surface to the posterior spinal canal at L5-S1, consistent with a dorsal dermal sinus (Fig. 1F and 1G). Based on clinical evaluation and MRI findings, a diagnosis of diastematomyelia Type II with associated hemimyelocele, tethered cord, filum terminale lipoma, and dorsal dermal sinus was established.

### Management

A neurosurgical consultation was obtained, and a thorough neurological examination revealed no focal neurological deficits. The patient was initially managed conservatively with nonsteroidal anti-inflammatory drugs for symptomatic relief of pain. After detailed evaluation, a surgical plan was formulated in view of radiological findings and anatomical abnormalities and discussed with the patient and his parties regarding the surgery and its outcomes. The surgical procedure included a duratomy, myelotomy, decompressive laminectomy, and excision of the bony spur, followed by duraplasty. Additionally, untethering of the filum terminale, interlaminar space dissection, and complete excision of the dermoid sinus tract were planned. These steps were aimed at relieving spinal cord tethering and preventing future neurological deterioration.

The patient and family were thoroughly counselled regarding the nature of the condition, the rationale for surgical intervention, expected outcomes, and potential complications. Detailed explanations of the surgical steps, the need for postoperative rehabilitation, and the importance of physiotherapy were provided. But the patient, being asymptomatic and knowing all the outcomes, cost of surgery, and rehabilitation duration, opted against the surgery, wanted to follow up regularly, and proceed with surgery upon further difficulties in the future. Thus, the patient was educated about danger signs, including worsening back pain, new-onset neurological symptoms, bowel or bladder dysfunction, and signs of infection, for which immediate medical attention would be necessary. Regular follow-up visits were scheduled to monitor neurological status, and neurology was intact with subsequent evaluations to date.

## Discussion

Diastematomyelia, or SCM, is a rare congenital spinal anomaly characterized by longitudinal division of the spinal cord into two hemi-cords [3]. The pathogenesis is believed to involve a defect in midline notochordal integration, leading to two notochordal processes that induce formation of separate neural plates [3,10]. SCM is classified into two types: Type I (diplomyelia), which accounts for 40%-50% of cases, features two hemi-cords, each within its own dural tube, separated by a rigid osseocartilaginous median septum [3,11]. Type II accounts for 50%-60% of cases and involves two hemi-cords within a single dural tube, separated by a fibrous intradural septum [3,11].

SCM is predominantly diagnosed in children and is rare in adults [11]. Delayed symptom onset in adults may be due to gradual spinal cord strain or traction on the conus medullaris [11,12]. Adult patients often lack skin or skeletal abnormalities common in pediatric cases [11]. Presentations may include neurological, orthopedic, or urological symptoms. However, some remain asymptomatic for years. In our case, a 23-year-old presented only with chronic back pain. A similar case was reported in a 72-year-old woman with the same symptom [13].

According to Chellathurai et al. [10], posterior myelomeningocele is the most common spinal dysraphism (14.2%), followed by posterior lipomyelomeningocele (3.33%), dermoid cysts (1.3%), and lumbar diastematomyelia (6%). Lipomeningocele occurs in approximately 0.6 per 10,000 live births, with half asymptomatic at birth [10]. Symptoms often develop progressively with spinal growth and rarely first present in adulthood [14].

Our case illustrates a highly complex form of spinal dysraphism, featuring Type II diastematomyelia, tethered cord due to filum terminale lipoma, a dorsal dermal sinus tract, and a neural-lipoma hemiplacode at the L5-S1 level. This rare combination highlights the intricacies of spinal cord development and the necessity for comprehensive diagnostics to identify and address all anomalies effectively.

TCS, a progressive condition seen in spinal dysraphism, arises from abnormal spinal cord attachment, either congenital (eg, diastematomyelia, lipomas) or postoperative (eg, meningocele repair) [6]. In our case, the cord was tethered by an intracanal sacral lipoma. Tethered cords may co-occur with lipomyelomeningocele and other abnormalities, including genitourinary tract anomalies (4.1%) and dermoid cysts (3.1%) [15]. Clinical presentation falls into three categories: neuro-orthopedic (sensory, motor, and trophic deficits, with pain common in adults), lumbosacral cutaneous (rare in adults), and sphincter dysfunction (in 80% of cases) [1,11]. Our patient’s primary symptom—chronic lower back pain—led to delayed diagnosis and subsequent neuroimaging.

Diagnosis of spinal dermoid cysts and diastematomyelia can be challenging due to subtle, nonspecific signs. Lipomyelomeningocele may be clinically suspected due to visible back swelling and neurologic symptoms [16]. While x-rays may be inconclusive, MRI is the preferred imaging modality for its superior soft tissue resolution and ability to delineate lesion characteristics [16]. MRI surpasses CT in evaluating spinal cord structure and adjacent soft tissues, making it essential for surgical planning [17]. Dermoid cysts typically appear on MRI as intraspinal masses with differential diagnoses including tumors, lipomas, and abscesses [16]. STIR MRI can help distinguish dermoid cysts from lipomas by identifying nonfat components [16]. Ultrasound, especially in the third trimester or in neonates, can assist in early detection of spinal anomalies and guide the need for further MRI evaluation [11].

Surgical intervention is generally the treatment of choice for diastematomyelia, regardless of symptoms [18]. However, patient-specific factors such as age, symptoms, and willingness to undergo surgery must guide management, especially in asymptomatic cases [13]. Vissarionov et al. [8] recommend basing decisions on deformity severity and progression. A bony spur is a common indication for surgery due to its link with progressive neurological decline [6,18]. Although additional procedures like untethering may be considered, their necessity depends on the severity of symptoms [6,18]. In our case, the patient, though advised surgery, opted for conservative management, preferring regular follow-up and reserving surgical intervention for future symptom progression.

Adult cases of Type II SCM with the constellation of anomalies seen in our patient—including hemimyelocele, filum terminale lipoma, tethered cord, and dorsal dermal sinus—are exceptionally rare. To better illustrate this, we performed a comprehensive literature review and summarized all previously reported adult cases with similar features in Table 1.

**Table 1:** Comprehensive review and summary of reviewed articles.

S.N.	Study	Patient/anomalies	Included features	Missing/different features
1	Malti [19]	50-y-old male: Type II diastematomyelia, tethered cord, filum terminale lipoma	Diastematomyelia, tethered cord, filum lipoma	No dermal sinus; no hemimyelocele
2	Hamidi et al. [1]	5-y-old boy: Type II diastematomyelia, tethered cord, intramedullary dermoid cyst, dermal sinus	Diastematomyelia, tethered cord, dermal sinus, dermoid cyst	No lipoma; no hemimyelocele
3	Awano and Wendimagegnehu [20]	2-y-old boy: Dermal sinus, dermoid cyst, filum lipoma, tethered cord, syringomyelia	Tethered cord, filum lipoma, dermal sinus, syrinx	No diastematomyelia; no hemimyelocele
4	Ak et al. [21]	Child: Type I diastematomyelia, tethered cord, filum lipoma, dermal sinus, epidermoid cyst	Diastematomyelia, tethered cord, filum lipoma, dermal sinus	Type I (not II); no hemimyelocele
5	Avcu et al. [8]	5-y-old boy: Tethered cord, syringomyelia, diastematomyelia, filum lipoma, epidermoid cyst, dermal sinus	Diastematomyelia, tethered cord, dermal sinus, filum lipoma, epidermoid, syrinx	Hemimyelocele not described
6	Dutta Satyarthee and Kumar [22]	11-y-old girl: Hemicord, dermal sinus, intramedullary dermoid cyst, fatty filum terminale	Hemicord (split cord), dermal sinus, dermoid, filum lipoma	No confirmation of Type II diastematomyelia; hemimyelocele-like only
7	Bothara et al. [23]	7-y-old girl: Type II diastematomyelia, spina bifida	Diastematomyelia	No sinus, no lipoma, no hemimyelocele
8	Hafeez et al. [24]	10-y-old child: Type II diastematomyelia, tethered cord, dorsal dermal sinus, lipomyelocele	Diastematomyelia Type II, tethered cord, dermal sinus, lipomatous lesion	Hemimyelocele not specified
9	Gupta et al. [25]	5-y-old boy: Type II diastematomyelia, tethered cord, terminal lipoma, spinal epidermoid, dermal sinus	Diastematomyelia, tethered cord, terminal lipoma, dermal sinus	Hemimyelocele not reported

The table highlights that most adult patients present with varied symptoms such as chronic back pain, neurological deficits, or urological complaints. Management approaches ranged from conservative follow-up to surgical intervention, with variable outcomes reported. Notably, none of the prior reports described the exact combination of anomalies found in our patient, underscoring the uniqueness of our case.

This comparative overview not only situates our findings within the existing literature but also reinforces the need for heightened clinical awareness and individualized management strategies in such complex presentations.

## Conclusion

This case presents a rare and complex form of spinal dysraphism in an adult patient who remained largely asymptomatic despite having multiple spinal anomalies, including Type II diastematomyelia, a tethered cord with filum terminale lipoma, a dorsal dermal sinus tract, and a neural-lipoma hemiplacode. The unique aspect of this case is the late presentation in adulthood with minimal symptoms, despite the extensive anatomical abnormalities, which underscores the highly variable clinical spectrum of such anomalies and emphasizes the pivotal role of high-resolution MRI in early detection, accurate diagnosis, and comprehensive surgical planning. Given the progressive nature of TCS and the risk of neurological deterioration, timely intervention is generally recommended. However, treatment decisions in asymptomatic adults should be individualized, taking into account patient preferences, potential risks, and the natural history of the condition. Given the rarity and complexity of such cases, a multidisciplinary approach involving neurosurgeons, radiologists, and rehabilitation specialists is indispensable for optimizing surgical planning, patient counseling, and long-term follow-up.

## Ethical approval

Case reports are exempt from ethical approval in our institution, Bir Hospital, Kathmandu.

## Availability of data and materials

The authors confirm that the data supporting the findings of this study are available within the article and Supplementary Files.

## Author contributions

ST and BK conceptualized and designed the study, drafted the initial manuscript, and reviewed and revised the manuscript. AG, RA, and JK designed the data collection instrument, collected data, carried out the initial analyses, and reviewed and revised the manuscript. AM and PM conceptualized and designed the study, coordinated and supervised data collection, and critically reviewed the manuscript for important intellectual content. All authors approved the final manuscript as submitted and agreed to be accountable for all aspects of the work.

## Patient consent

Written informed consent was obtained from the patient for participation and publication of this case reports and accompanying images. A copy of the written consent is available for review by the Editor-in-Chief of this journal on request.
